# Pandemics in the Age of Twitter: Content Analysis of Tweets during the 2009 H1N1 Outbreak

**DOI:** 10.1371/journal.pone.0014118

**Published:** 2010-11-29

**Authors:** Cynthia Chew, Gunther Eysenbach

**Affiliations:** 1 Centre for Global eHealth Innovation, University Health Network, Toronto, Canada; 2 Department of Health Policy, Management and Evaluation, Faculty of Medicine, University of Toronto, Canada; Children's Hospital of Eastern Ontario, Canada

## Abstract

**Background:**

Surveys are popular methods to measure public perceptions in emergencies but can be costly and time consuming. We suggest and evaluate a complementary “infoveillance” approach using Twitter during the 2009 H1N1 pandemic. Our study aimed to: 1) monitor the use of the terms “H1N1” versus “swine flu” over time; 2) conduct a content analysis of “tweets”; and 3) validate Twitter as a real-time content, sentiment, and public attention trend-tracking tool.

**Methodology/Principal Findings:**

Between May 1 and December 31, 2009, we archived over 2 million Twitter posts containing keywords “swine flu,” “swineflu,” and/or “H1N1.” using Infovigil, an infoveillance system. Tweets using “H1N1” increased from 8.8% to 40.5% (*R* ^2^ = .788; *p*<.001), indicating a gradual adoption of World Health Organization-recommended terminology. 5,395 tweets were randomly selected from 9 days, 4 weeks apart and coded using a tri-axial coding scheme. To track tweet content and to test the feasibility of automated coding, we created database queries for keywords and correlated these results with manual coding. Content analysis indicated resource-related posts were most commonly shared (52.6%). 4.5% of cases were identified as misinformation. News websites were the most popular sources (23.2%), while government and health agencies were linked only 1.5% of the time. 7/10 automated queries correlated with manual coding. Several Twitter activity peaks coincided with major news stories. Our results correlated well with H1N1 incidence data.

**Conclusions:**

This study illustrates the potential of using social media to conduct “infodemiology” studies for public health. 2009 H1N1-related tweets were primarily used to disseminate information from credible sources, but were also a source of opinions and experiences. Tweets can be used for real-time content analysis and knowledge translation research, allowing health authorities to respond to public concerns.

## Introduction

*“In the era of the 24-hour news cycle, the traditional once-a-day press conference featuring talking heads with a bunch of fancy titles has to be revamped and supplemented with Twitter posts, YouTube videos and the like. The public needs to be engaged in conversations and debate about issues of public health, they don't need to be lectured to.”*

-Andre Picard [1]

Public health agencies do not act in a void, but rather are part of a larger feedback loop that includes both the media and the public. The social amplification of risk framework postulates that psychological, social, cultural, and institutional factors interact with emergency events and thereby intensify or attenuate risk perceptions [2]. Traditionally, print media, TV and radio are the major transmitters of information from public health agencies to the public and play a large role in risk intensification and attenuation. However, during the most recent public health emergency, 2009 H1N1, respondents cited the internet as their most frequently used source of information on the pandemic [3].

With the rise of the participatory web and social media (“Web 2.0”) and resulting proliferation of user-generated content, the public potentially plays a larger role in all stages of knowledge translation, including information generation, filtering, and amplification. Consequently, for public health professionals, it is increasingly important to establish a feedback loop and monitor online public response and perceptions during emergency situations in order to examine the effectiveness of knowledge translation strategies and tailor future communications and educational campaigns.

Surveys are the traditional methods for public health officials to understand and measure public attitudes and behavioural responses. Several studies have used telephone, internet, and in-person surveys to elicit such information during the H1N1 pandemic (e.g., [4], [5]). Rapid-turnaround surveys best capture changes in attitudes and behaviour influenced by specific events and produce the most relevant information for agency intervention [6]. Unfortunately, time is needed to gather resources, funding, and survey instruments for polling [6].

New “infoveillance” methods such as mining, aggregating, and analysing online textual data in real-time are becoming available [7], [8]. Twitter (www.twitter.com) is potentially suitable for longitudinal text mining and analysis. The brief (<140 characters) text status updates (“tweets”) users share with “followers” (e.g., thoughts, feelings, activities, opinions) contain a wealth of data. Mining these data provides an instantaneous snapshot of the public's opinions and behavioural responses. Longitudinal tracking allows identification of changes in opinions or responses. In addition to quantitative analysis, the method also permits qualitative exploration of likely reasons *why* sudden changes have occurred (e.g., a widely read news report) and may indicate what is holding the public's attention [9].

H1N1 marks the first instance in which a global pandemic has occurred in the age of Web 2.0 and presents a unique opportunity to investigate the potential role of these technologies in public health emergencies. Using an “infoveillance” approach we report on: 1) the use of the terms “H1N1” versus “swine flu” over time on Twitter, to establish the feasibility of creating metrics to measure the penetration of new terms and concepts (knowledge translation), 2) an in-depth qualitative analysis of tweet content, expression, and resources, and 3) the feasibility and validation of using Twitter as a real-time content, sentiment, and public attention trend-tracking tool.

## Methods

### Data Collection & Database

We developed an open-source infoveillance system, Infovigil [8], which continuously gathered and mined textual information from Twitter via its Application Programming Interface (the original Twitter data is available at http://infovigil.com/). Every few seconds, Infovigil gathered new publically-available tweets containing keywords of interest and stored them in an internal relational database, including metadata such as username and time. Between May 1 and December 31, 2009, we archived over 2 million tweets containing keywords or hashtags (#) “H1N1”, “swine flu”, and “swineflu”. In addition to recording tweets, we archived the cited web pages beginning in September 2009. This database served as the primary dataset for our study. All statistical analyses used SPSS.

### 1) Knowledge Translation: H1N1 vs. Swine Flu Terminology

To establish a knowledge translation metric to measure the terminology shift from colloquial term “swine flu” to World Health Organization (WHO)-recommended “H1N1” [10], a linear regression for the proportion of tweets citing “H1N1” over time was performed using English-only tweets from May 1 to December 31, 2009. Tweets utilizing both “swine flu” and “H1N1” were counted toward the overall total but not the proportion of H1N1 or swine flu tweets.

### 2) Manual Content & Sentiment Analysis

Qualitative manual coding of tweets commenced on Monday, May 11, 2009, the first set of complete data available. To look for changing content at systematic periods, Mondays, 4 weeks apart were selected over the remainder of 2009 (total of 9 days). Because we were mainly interested in trends, we held the day of the week constant to avoid artificial peaks caused by sampling from different days of the week. 25 randomly selected tweets from every hour of the aforementioned days were coded to avoid time bias associated with posting. Since there are no prior methodologies for sampling tweets, we were unable to perform a formal sample size calculation. Instead, we chose our sample size based on feasibility and determined that 25 tweets per hour (600 tweets per day) would be sufficient to capture a daily “snapshot”. Any re-posted or “re-tweeted” tweets using notation “RT @ username” or “RT@username” were excluded to prevent popular posts or spam from saturating the sample. Non-English tweets were also excluded because translation was not feasible.

#### Codebook

We created a tri-axial coding scheme using an iterative process to reflect: 1) the tweet's content, 2) how it was expressed, and 3) the type of link posted, if any. Preliminary coding of 1200 tweets provided the initial categories and codebook. Upon review and discussion, infrequently used categories were collapsed into larger concepts and a subset of tweets (125) was coded by two raters to establish coding reliability (kappa). The last iteration of the codebook was finalized when a sufficient kappa level (>0.7) was obtained for each axis of the coding scheme.

Where multiple qualifiers were present within a tweet, all applicable qualifiers were used. Neutral or ambiguous statements were not coded. Tweets were categorized as misinformation if the tweet was not categorized as a joke and was unsubstantiated by our reference standards: the Centers for Disease Control (CDC) and Public Health Agency of Canada for scientific claims and a panel of credible online news sources (e.g., CNN, BBC) for news-related claims.

#### Analysis

The chi-square test for trend was used to determine if the proportion of content, qualifiers, or links tweeted changed linearly over our analysis timeframe. Prior to testing for linearity, scatterplots were performed on each category to detect any non-linear patterns.

### 3) Automated Content & Sentiment Analysis

#### Query Development

Infovigil was configured for real-time analysis and visualization of the tweets by constructing SQL (Structured Query Language) queries searching for keywords and phrases that matched our content categories. For validation purposes, and to maintain consistency with manual coding, we filtered out retweets, i.e. tweets containing “RT@” or “RT @”. While tweet searches included data from May 1, 2009 to the present day, we only used data from the 9 selected days as comparison points with the manual coding. All qualifiers along with 3 content categories (resources, personal experiences and personal opinions/interest) were transformed into concept queries. Initial search patterns (keywords or phrases) for each concept were derived from the codebook and an ongoing list of common phrases. Common misspellings, emoticons, internet slang, and keyword variants were also included. Keywords were modified to include/exclude specified prefixes. Results from each keyword were audited to estimate its precision. Audits were conducted by viewing the results for each keyword for three randomly selected days. Search patterns were modified or deleted if approximately more than 30% of tweets did not reflect the concept.

#### Validation Analysis

Concept query totals from the 9 selected days were recorded. Pearson's correlations were used to measure the relationship between the proportions of selected categories resulting from the manual coding and the automated analyses. Automated proportions were obtained by taking the amount of tweets that were returned by a concept search query (e.g., tweets labelled as “personal experience”) and dividing by the total amount of tweets per day. Additionally, chi-square tests for trend were used to determine if changes in automated concepts were trending similar to the manual coding. To conduct external validation, both the proportion and absolute number of weekly automated tweets sharing personal experiences and concern were compared to weekly US H1N1 incidence rates from WHO's FluNet reporting tool (http://gamapserver.who.int/GlobalAtlas/home.asp) using Pearson's correlations. We expected that these two types of tweets would be positively correlated with incidence rates. US incidence rates were chosen because Americans account for the largest proportion of Twitter users [11].

#### Public Attention on Twitter

Longitudinal results from the automated queries were automatically graphed by Infovigil. These graphs were visually examined for large spikes in tweet volume (a potential indicator of public attention) and tweets on those days were reviewed to see what media stories or external events influenced these peaks. For clarity, the largest peak in each longitudinal graph was scaled to 100 on the y-axis and all other peaks were plotted relative to that peak.

### 4) Audit of Retweets

Both our manual and automated analysis excluded retweets (RTs). As RTs may be systematically different from original or non-retweets (nonRTs), we performed a sub-analysis on RTs. 3 RTs from every hour of the 9 selected days (12% of the manual sample) were manually coded using the same methodology described previously. Chi-square tests were used to observe differences between manually coded RTs and nonRTs. Fisher's exact tests were used when cell counts were less than 5. Chi-square tests for trend were used to detect linear trends over time. Trend results for RTs were compared to trends of nonRTs.

To compare RTs and nonRTs from the automated analysis, queries for each of the 10 concepts were modified to include RTs in the search results. Longitudinal graphs of RT and nonRT results for each automated query concept were compared visually. Noticeable differences in graph shape or spike volume were noted.

## Results

### 1) Knowledge Translation: H1N1 vs. Swine Flu Terminology

**Figure 1:**
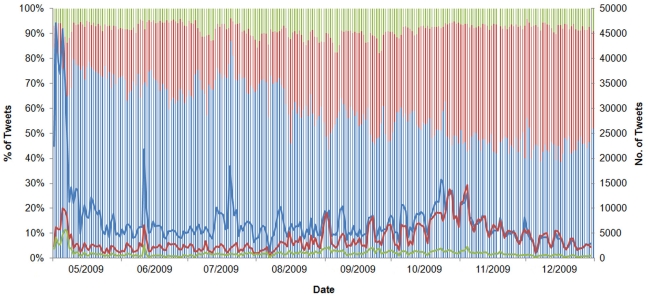
Tweets containing H1N1, swine flu, or both from May to December 2009. Lines = absolute number. Bars = relative percentage. Blue = “swine flu” or swineflu. Red = H1N1. Green = (“swine flu” or swineflu) AND H1N1.

Between May 1 and December 31, 2009, the relative proportion of tweets using “H1N1” increased from 8.8% to 40.5% in an almost linear fashion (*R* ^2^ = .788; *p*<.001), indicating a gradual adoption of the WHO-recommended H1N1 terminology as opposed to “swine flu” (Figure 1). “H1N1” use became equally prevalent as “swine flu” use on September 1.

### 2) Manual Content & Sentiment Analysis

#### Coding Scheme

Six content categories emerged from the data: resources, direct or indirect personal experiences (e.g., “I have swine flu”), personal reactions to or opinions (e.g., “I'm scared of H1N1”), jokes/parodies, marketing for H1N1-related products, and unrelated posts (Table 1).

**Table 1:** Descriptions and Examples of Content Categories.

Content	Description	Example Tweets
Resource	Tweet contains H1N1 news, updates, or information. May be the title or summary of the linked article. Contents may or may not be factual.	*“China Reports First Case of Swine Flu (New York Times): A 30-year-old man who flew from St. Louis to Chengdu is.. * http://tinyurl.com/rdbhcg *”* *“Ways To Prevent Flu * http://tinyurl.com/r4l4cx * #swineflu #h1n1”*
Personal Experience	Twitter user mentions a direct (personal) or indirect (e.g., friend, family, co-worker) experience with the H1N1 virus or the social/economic effects of H1N1.	*“Swine flu panic almost stopped me from going to US, but now back from my trip and so happy I went :-))”* *“Oh we got a swine flu leaflet. clearly the highlight of my day”* *“My sister has swine flu!”*
Personal Opinion and Interest	Twitter user posts their opinion of the H1N1 virus/situation/news or expresses a need for or discovery of information. General H1N1 chatter or commentary.	*“More people have died from Normal Flu than Swine flu, its just a media hoax, to take people's mind off the recession”* *“Currently looking up some info on H1N1”* *“Swine flu is scary!”*
Jokes/Parody	Tweet contains a H1N1 joke told via video, text, or photo; or a humourous opinion of H1N1 that does not refer to a personal experience.	*“If you're an expert on the swine flu, does that make you Fluent?”*
Marketing	Tweet contains an advertisement for an H1N1-related product or service.	*“Buy liquid vitamin C as featured in my video * http://is.gd/y87r * #health #h1n1”*
Spam	Tweet is unrelated to H1N1	*“musicmonday MM lamarodom Yom Kippur Polanski Jay-Z H1N1 Watch FREE online LATEST MOVIES at * http://a.gd/b1586f *”*

Tweets not resource or spam-based were coded with a qualifier, if present. The codebook definitions of the 7 qualifiers took into consideration specific keywords and phrases, common internet expressions (e.g., “lol”), and emoticons (textual expressions representing a face or mood) (Table 2). Tweeted URLs were categorized into one of nine categories (Table 3).

**Table 2:** Descriptions and Examples of Qualifier Categories.

Qualifier	Description	Example Emoticons or Internet Slang	Example Tweets
Humour or Sarcasm	Tweet is comedic or sarcastic.	:) ;) :-) :PLOL, ROFL, j/k	*“Rupert Grint had Swine Flu. It's VOLDEMORTS COMEBACK!”* *“babysitting kids with h1n1, awesome. cant wait til thursday!!”*
Relief	Tweet expresses joy, happiness, or sense of peace.	:) = ) :D	*“in Egypt….without the swine flu…YAY!”* *“thank God my mom and bro don't have that swine flu it was just a cold”*
Downplayed Risk	Tweet attempts to de-emphasize the potential risk of H1N1 or bring it into perspective. May also express a lack of concern or disinterest.	-_-	*“90 ppl get swine flu & everyone wants to wear surgical masks. 1 million ppl have aids & nobody wants to wear a condom”* *“everyone needs to calm down about swine flu. it'll all be ok.”*
Concern	Tweet expresses H1N1-related fear, anxiety, worry, or sadness for self or others. May also express scepticism.	:( :`( :\ :SOMG, r u ok?	*“Hope the girl sitting on the tram next to me doesn't have swine flu. She is sneezing all over the place”* *“my baby sister has the swine flu. :(”* *“omg i know someone who has the swine flu .. its so sad”*
Frustration	Tweet expresses anger, annoyance, scorn, or volatile contempt. May include coarse language.	>:| >:(FML, grr	*“If I hear another DAMN thing about the recession and swine flu I swear Ima hurt somebodys feelings!”* *“i hate swine flu!!!!!! it ruins everything i tell yeah!!!!!”*
Misinformation	Tweet contradicts the reference standard or contains unsubstantiated information. May make speculations or express distrust of authority or the media. May include conspiracy or doomsday theories.		*“Swine Flu Vaccine will kill you! * http://tinyurl.com/ph8sgx *”* *“The swine flu is pharmaceutical conspiracy, a way to quick money”*
Question	Tweet asks a question or contains a question mark.		*“Why is it actually called #swineflu and not pigflu or hogflu?”*

**Table 3:** Descriptions and Examples of Link Categories.

Link	Description	Example Webpage
Mainstream or Local News	Webpage links to a local or international TV/radio/print/internet news service.	www.CNN.com www.BBC.co.uk www.Reuters.com
News Blog, Feed, or Niche News	Webpage primarily provides aggregated news content, news briefs, or has a specialized news focus. Webpage may allow for user-submitted articles to be published.	www.H1N1Alliance.com www.MedicalNewsToday.com www.SoccerNet.com
Government or Public Health	Webpage of a government or public health authority. Health professionals associations and private health organizations are excluded.	www.CDC.gov www.WHO.int
Personal Blog	Personal webpage or blog that the user may post thoughts, opinions, or experiences.	www.blogger.com www.wordpress.com
Social Network	Webpage links to a social networking page, such as a user's status update.	www.Facebook.com www.MySpace.com www.Twitter.com
Online Store	Webpage links to a store or advertisement.	www.ebay.com www.Purell.com
Other	Webpage is not described above. May be centered on providing services, editorials, media, or reference material.	www.YouTube.com www.PrisonPlanet.com www.About.com
No Reference	Tweet made claims or presented information without providing a URL	
Not Accessible	URL is not accessible	

#### Content Analysis

We analyzed 5,395 tweets for our content analysis (Table 4). The total number of tweets was short by 5 because we did not gather enough eligible tweets on September 28 for analysis. The inter-rater reliability (kappa) was estimated as 0.80 for content, 0.74 for qualifiers, and 0.84 for links. H1N1 resources were the most common type of content shared (52.6%), followed by personal experiences (22.5%). 39% of tweets were coded with 1 or more qualifiers. Tweets expressing humour (12.7%), concern (11.7%), and questions (10.3%) were the most common, while 4.5% were classified as possible misinformation. 61.8% of all tweets had links, 23.2% of all posts linked to a news website, while links to government and public health agencies were not commonly shared (1.5%). 90.2% of tweets provided links when a reference was necessary.

**Table 4:** Content, Qualifiers, and Links of Manually Coded H1N1 Tweets.

	------------------------ Type of Tweets, No. (%) ------------------------										
	May 11(n = 600)	June 8(n = 600)	July 6(n = 600)	Aug 3(n = 600)	Aug 31(n = 600)	Sept 28^a^(n = 595)	Oct 26(n = 600)	Nov 23(n = 600)	Dec 21(n = 600)	Total (5395)	*p* Value, trend
**Content**											
Resources	291 (49)	330 (55)	252 (42)	284 (47)	352 (59)	302 (51)	296 (49)	366 (61)	367 (61)	2840 (53)	**<.001** *
Personal Experiences	107 (18)	119 (20)	140 (23)	150 (25)	94 (16)	158 (27)	176 (29)	138 (23)	132 (22)	1214 (23)	**.01** *
Personal Opinion	81 (14)	80 (14)	147 (25)	93 (16)	76 (13)	82 (14)	78 (13)	51 (9)	52 (9)	740 (14)	**<.001** *
Jokes	100 (17)	53 (9)	50 (8)	52 (9)	45 (8)	28 (5)	28 (5)	30 (5)	35 (6)	421 (8)	**<.001** *
Marketing	7 (1)	10 (2)	6 (1)	15 (3)	10 (2)	2 (0)	2 (0)	9 (2)	11 (2)	72 (1)	.72
Spam	14 (2)	8 (1)	5 (1)	6 (1)	23 (4)	23 (4)	20 (3)	6 (1)	3 (1)	108 (2)	.91
**Qualifiers** b											
Humour	129 (22)	81 (14)	72 (12)	87 (15)	59 (10)	65 (11)	63 (11)	58 (10)	73 (12)	687 (13)	**<.001** *
Relief	13 (2)	3 (1)	6 (1)	10 (2)	7 (1)	5 (1)	15 (3)	9 (2)	13 (2)	81 (2)	.18
Downplayed Risk	17 (3)	14 (2)	10 (2)	19 (3)	7 (1)	7 (1)	20 (3)	11 (2)	1 (0)	106 (2)	**.014** *
Concern	47 (8)	57 (10)	105 (18)	85 (14)	69 (12)	81 (14)	76 (13)	57 (10)	56 (9)	633 (12)	.677
Frustration	25 (4)	27 (5)	29 (5)	32 (5)	23 (4)	33 (6)	18 (3)	13 (2)	12 (2)	212 (4)	**.002** *
Misinform.	23 (4)	13 (2)	13 (2)	55 (9)	37 (6)	34 (6)	28 (5)	26 (4)	14 (2)	243 (5)	.756
Question	66 (11)	60 (10)	76 (13)	59 (10)	63 (11)	47 (8)	84 (14)	49 (8)	51 (9)	555 (10)	.125
**Links** c											
News Websites	141 (24)	159 (27)	119 (20)	142 (24)	172 (29)	140 (24)	138 (23)	126 (21)	116 (19)	1253 (23)	**.04** *
News Blogs, Feeds, or Niche News	72 (12)	76 (13)	40 (7)	62 (10)	76 (13)	47 (8)	69 (12)	79 (13)	111 (19)	632 (12)	**.001** *
Government or Public Health	9 (2)	8 (1)	11 (2)	9 (2)	5 (1)	10 (2)	11 (2)	8 (1)	10 (2)	81 (2)	.826
Personal Blogs	8 (1)	14 (2)	20 (3)	7 (1)	11 (2)	5 (1)	16 (3)	12 (2)	6 (1)	99 (2)	.348
Social Networks	6 (1)	13 (2)	9 (2)	12 (2)	6 (1)	19 (3)	26 (4)	19 (3)	22 (4)	132 (2)	**<.001** *
Online Stores	12 (2)	1 (0)	7 (1)	17 (3)	8 (1)	1 (0)	1 (0)	6 (1)	13 (2)	66 (1)	.668
Other	39 (7)	33 (6)	41 (7)	48 (8)	75 (13)	73 (12)	45 (8)	92 (15)	64 (11)	510 (10)	**<.001** *
No Reference	23 (4)	56 (9)	78 (13)	50 (8)	32 (5)	26 (4)	19 (3)	25 (4)	17 (3)	326 (6)	**<.001** *
Not Accessible	37 (6)	30 (5)	13 (2)	14 (2)	29 (5)	20 (3)	14 (2)	33 (6)	45 (8)	235 (4)	.20

a 2300 hrs on Sept. 28 had only 20 eligible tweets.

b The percent total will not equal 100% as not all tweets had qualifiers and tweets with multiple qualifiers were coded multiple times. Approximately 40% of tweets contained qualifiers.

c The total will not equal to 100% as not all tweets had links. Approximately 60% of tweets contained links.

*chi-square test for trend significant at *p*<0.05.

**Figure 2:**
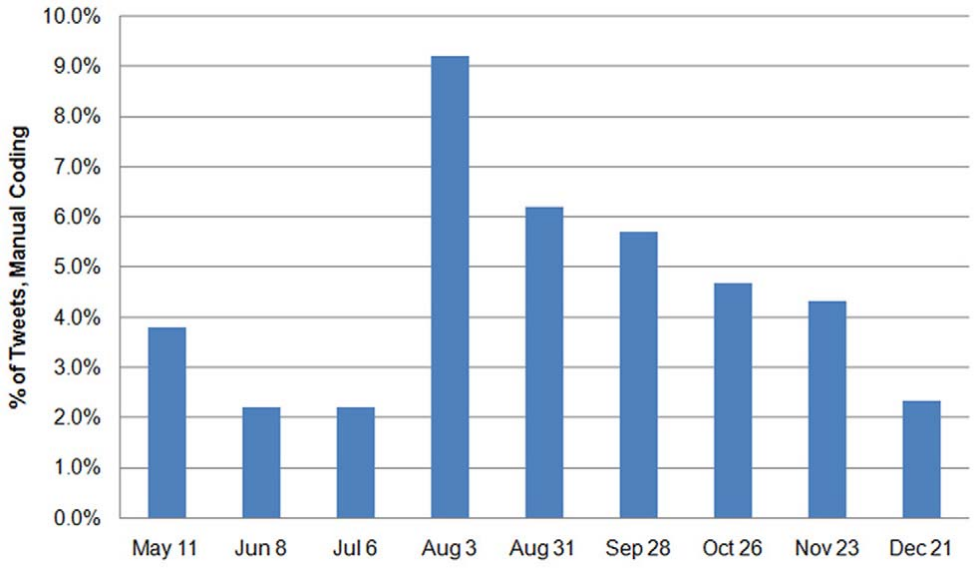
Non-linear pattern of tweeted misinformation identified via manual coding from May to December 2009.

The chi-square test for trend showed several linear trends in the data (Table 4). The proportion of tweets containing resources and personal experiences increased over time, while the amount of jokes and personal opinions/interest decreased. Tweets expressing humour, frustration, and downplayed risk became less common. Mainstream and local news websites were cited significantly less, while references to news blogs/feeds/niches, social networks, and other web pages increased. No significant trends were found for misinformation, but the data exhibited a non-linear pattern (Figure 2).

### 3) Automated Content & Sentiment Analysis

#### Validation

**Figure 3:**
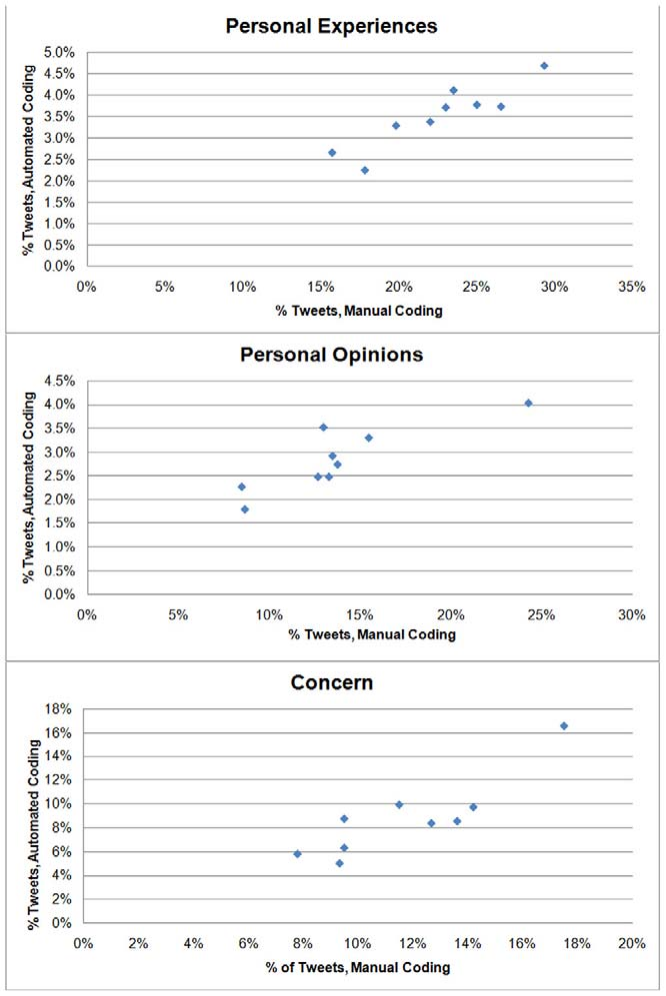
Example scatterplots of manual versus automated coding proportions.

**Figure 4:**
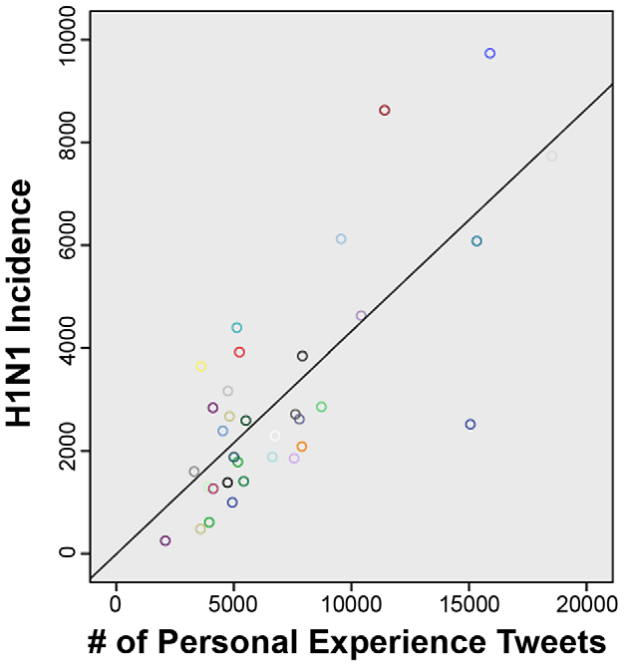
Scatterplot of tweets sharing personal experiences and USA H1N1 incidence rate.

**Figure 5:**
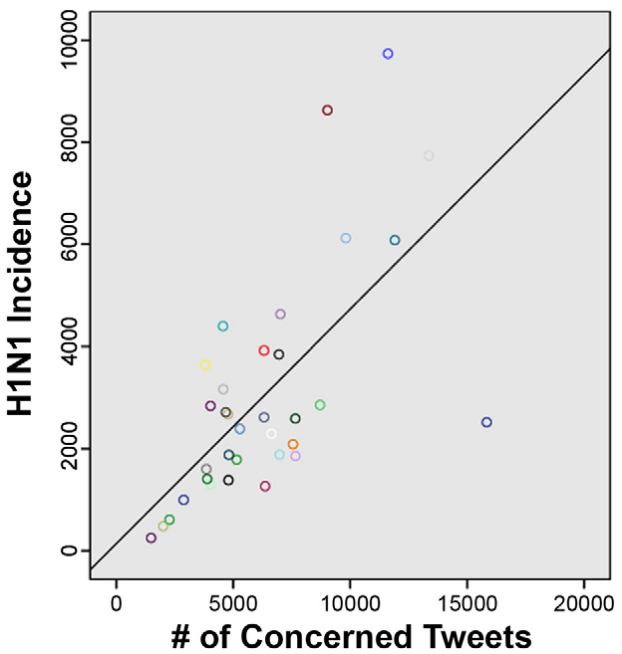
Scatterplot of tweets expressing concern and USA H1N1 incidence rate.

Table 5 presents examples of search patterns used to develop queries (see Table S1 for full list of detailed queries). 7/10 automated queries were found to correlate significantly with the results of manual coding (Table 6), including personal experiences (*r* = 0.91), concern (*r* = 0.87), and personal opinion/interest (*r* = 0.86) (Figure 3). H1N1 incidence rates were correlated with the absolute number of tweets sharing personal experiences (*r* = .77, *p*<.001) (Figure 4) and concern (*r* = .66, *p*<.001, Figure 5), as did the percentage of tweets sharing personal experiences (*r* = .67, *p*<.001) and concern (*r* = 0.39, *p* = .02).

**Table 5:** Descriptions and Examples of Automated Coding Search Patterns.

Concept	Example Keywords	Example Emoticons	Example Phrases
Humour/Sarcasm	lolhahaj/k	:P	“when pigs fly”“just kiddin”“bacon flu”
Concern	omgafraidworried	:(	“freaking out”“uh oh”“stay away”
Frustration	annoystupidangry	>:|	“swine flu sucks”“hate swine flu”“so sick of”
Downplayed Risk	overblownhypehysteria		“calm down”“not a big deal”“forget about swine flu”
Relief	whewgratefulthankful		“thank God”“glad to hear”“feeling better”
Misinformation	conspiracytoxinautism		“mind control”“chemical warfare”“real story”
Personal Experiences	my (mom, co-worker, classmate, teacher, etc.)		“went to get my swine flu shot”“feeling sick”“going to a clinic”
Personal Opinions/Interest	imhoheardreading		“I believe”“government should”“I feel that”
Resources	http://https://		
Questions	?		

**Table 6:** Correlations between Manual and Automated Coding.

Concept	Pearson Correlation (*r*)	*p* value
Resources	.83	**.006** *
Personal Experiences	.91	**.001** *
Personal Opinion/Interest	.86	**.003** *
Humour/Sarcasm	.70	**.037** *
Relief	−.38	.312
Downplayed Risk	.08	.845
Concern	.87	**.002** *
Frustration	.45	.228
Misinformation	.815	**.007** *
Question	.83	**.005** *

N = 9.

*significant at *p*<0.05.

Chi-square tests for trend found that all 3 content concepts and 4 of 7 qualifier concepts displayed significant linear trends over our timeframe (Table 7). The content categories all trended in the same direction as in the manual coding. Humour/sarcasm and downplayed risk trends also had the same downward trends as in the manual analysis. Trends for misinformation and concern were unique to the automated coding. Although a downward trend for frustration was found in the manual coding, no such pattern was observed in the automated analysis.

**Table 7:** Automated Coding Trends over Time.

Concept	χ^2^ test for trend (*df* = 1)	P value	Automated coding trend over time	Manual coding trend over time
Resources	252.98	**.000** *		
Personal Experience	73.83	**.000** *		
Personal Opinion & Interest	6.42	**.011** *		
Humour & Sarcasm	292.54	**.000** *		
Relief	.41	.522		
Downplayed Risk	7.85	**.005** *		
Frustration	2.64	.104		
Concern	25.47	**.000** *		
Misinformation	13.66	**.000** *		
Question	.51	.477		

*chi-square test for trend significant at *p*<0.05.

#### Public Attention on Twitter

**Figure 6:**
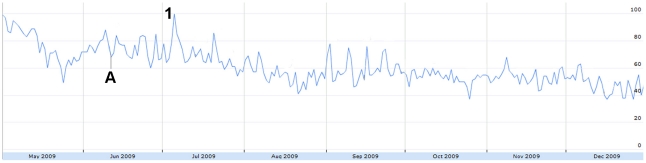
The relative proportion of tweets expressing humour from May 1 to December 31, 2009. Figure was scaled to the highest peak on July 5 where 9.82% of all tweets expressed misinformation. The peak was assigned a score of 100. A = June 11: WHO pandemic level 6 announcement. 1 = July 5: Harry Potter actor Rupert Grint has H1N1.

**Figure 7:**
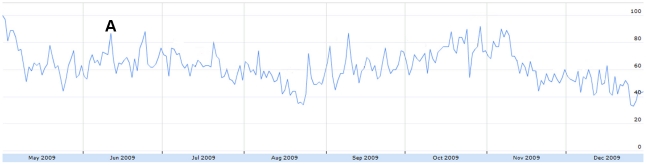
The relative proportion of tweets expressing frustration from May 1 to December 31, 2009. Figure was scaled to the highest peak on May 1 where 4.65% of all tweets expressed frustration. The peak was assigned a score of 100. A = June 11: WHO pandemic level 6 announcement.

Sharp increases in absolute H1N1-related tweet volume coincided with major H1N1 news events. For example, a large peak on June 11 (Figure 1) corresponded to the WHO's Pandemic Level 6 announcement [12]. The volume of humorous tweets also decreased on this day (Figure 6) and the number of frustrated tweets increased (Figure 7).

**Figure 8:**
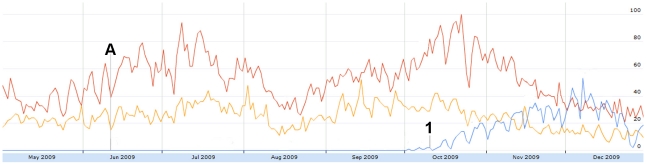
The relative proportion of tweets sharing personal experiences from May 1 to December 31, 2009. Figure was scaled to the highest peak on Oct. 20 where 5.64% of all tweets shared personal experiences. The peak was assigned a score of 100. Subconcepts: Red = indirect (family/friend) experience. Yellow = personal/direct experience. Blue = vaccination experience. A = June 11: WHO pandemic level 6 announcement. 1 = Oct 6: H1N1 vacctionations arrive in the US.

In Figure 8, the October to November peak directly coincides with the second wave of H1N1 in North America [13]. Similarly, when personal experiences were further broken down into sub-concepts, tweet volume of vaccination experiences increased rapidly following the arrival of H1N1 vaccinations in the United States on October 6 [14].

**Figure 9:**
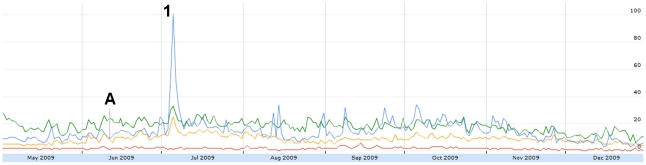
The relative proportion of tweets expressing concern from May 1 to December 31, 2009. Figure was scaled to the highest peak on July 5 where 30.66% of all tweets expressed concern. The peak was assigned a score of 100. Subconcepts: Blue = concern for others. Red = concern for self. Yellow = concerned emoticons. Green = general concern. A = June 11: WHO pandemic level 6 announcement. 1 = July 5: Harry Potter actor Rupert Grint has H1N1.

**Figure 10:**
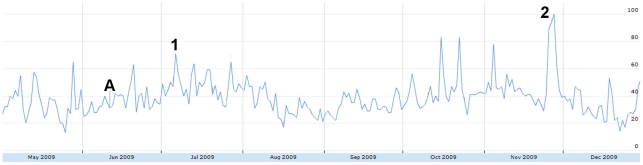
The relative proportion of tweets expressing relief from May 1 to December 31, 2009. Figure was scaled to the highest peak on Nov. 27 where 1.33% of all tweets expressed relief. The peak was assigned a score of 100. A = June 11: WHO pandemic level 6 announcement. 1 = July 5: Harry Potter actor Rupert Grint has H1N1. 2 = Nov 25–27: #iamthankfulfor campaign.

Tweets expressing concern had one outstanding peak on July 5 (Figure 9), coinciding with a news story that one of the actors from “Harry Potter” was recovering from H1N1 [15]. Humour (Figure 6) and relief (Figure 10) also increased in response to this story.

**Figure 11:**
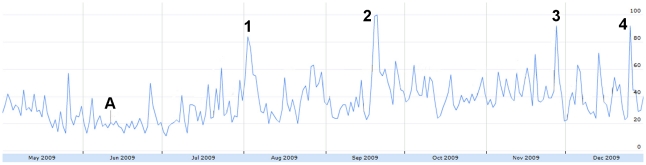
The relative proportion of tweets expressing misinformation from May 1 to December 31, 2009. Figure was scaled to the highest peak on Sept. 20 where 1.93% of all tweets expressed misinformation. The peak was assigned a score of 100. A = June 11: WHO pandemic level 6 announcement. 1 = Aug 2: CBS reports on parental concerns about H1N1. 2 = Sept 18–21: Ten swine flu lies told by the mainstream media. 3 = Nov 27: WHO and drug companies in collusion. 4 = Dec 25: Carbon monoxide poisoning can create same symptoms as H1N1.

Misinformation displayed several large peaks in our timeframe (Figure 11). The largest peak appeared from September 18–21 with circulation of a story listing the “ten swine flu lies told by the mainstream media” [16]. Other peaks (August 2, December 25) were not the result of true misinformation or speculation, rather the popular news stories on those days had keywords associated with the misinformation query [17], [18].

**Figure 12:**
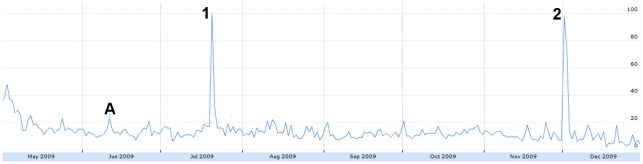
The relative proportion of tweets expressing downplayed risk from May 1 to December 31, 2009. Figure was scaled to the highest peak on July 20 where 6.96% of all tweets expressed downplayed risk. The peak was assigned a score of 100. A = June 11: WHO pandemic level 6 announcement. 1 = July 20: Viral dissemination of the “face mask (H1N1) versus condom (AIDS) comparison.” 2 = Dec 1: Viral dissemination of the “face mask (H1N1) versus condom (AIDS) comparison.”

**Figure 13:**
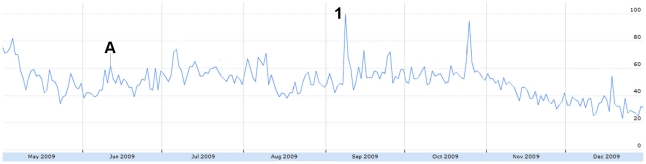
The relative proportion of tweets sharing personal opinions & interest from May 1 to December 31, 2009. Figure was scaled to the highest peak on Sept. 8 where 5.34% of all tweets shared personal opinions. The peak was assigned a score of 100. A = June 11: WHO pandemic level 6 announcement. 1 = Sept. 8: Case of H1N1 confirmed at PAX videogame convention in Seattle.

Viral dissemination of campaigns on Twitter resulted in several large spikes. One campaign comparing the perceived need for face masks for H1N1 to condoms for AIDS was responsible for two large peaks in “downplayed risk” on July 20 and December 1 (Figure 12). The “#iamthankfulfor” campaign, taking place between November 25–27 (American Thanksgiving), resulted in the largest peak of tweets expressing relief. In this campaign, users posted items they were thankful for, which in our data was related to getting the H1N1 vaccine or not becoming infected. Another notable campaign was the “#oink” movement on August 16 to support the pork industry and farmers by urging the media and public to use “H1N1” instead of “swine flu” [19]. Consequently, the number tweets using “H1N1” increased and those using “swine flu” decreased. In one case, viral dissemination of new information caused a large activity spike of tweets (Figure 13). On September 8, Twitter was used to report the discovery of the first confirmed H1N1 case at a videogame convention in Seattle and urged symptomatic attendees to seek medical advice [20].

**Figure 14:**
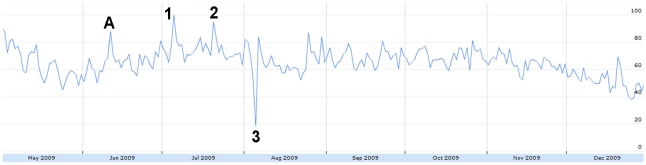
The relative proportion of tweets sharing questions from May 1 to December 31, 2009. Figure was scaled to the highest peak on July 5 where 18.99% of all tweets shared questions. The peak was assigned a score of 100. A = June 11: WHO pandemic level 6 announcement. 1 = July 5: Harry Potter actor Rupert Grint has H1N1. 2 = July 20: Viral dissemination of the “face mask (H1N1) versus condom (AIDS) comparison.” 3 = August 5: Unexplained drop in questions.

**Figure 15:**
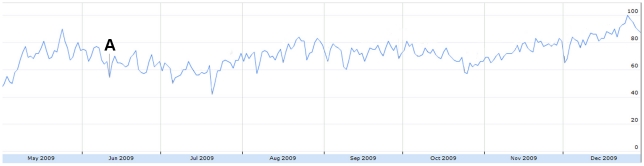
The relative proportion of tweets sharing resources from May 1 to December 31, 2009. Figure was scaled to the highest peak on Dec. 25 where 77.27% of all tweets shared resources. The peak was assigned a score of 100. A = June 11: WHO pandemic level 6 announcement.

The largest volume of questions posted on Twitter coincided with the WHO pandemic level 6 announcement (June 11), the “Harry Potter” actor illness (July 5), and the face mask versus condom campaign (July 20) (Figure 14). An unexplained significant drop in questions occurred on August 5. An upward trend without major peaks was found within tweeted resources (Figure 15).

### 4) Retweet Analysis

Manual coding of RTs found that the proportion of tweets sharing personal experiences was significantly less compared to nonRTs (χ^2^(1) = 11.45, *p* = .001). No other significant differences in aggregated data were found. Chi-square test for trend found significant downward trends for jokes (χ^2^(1) = 6.83, *p* = .009) and humour (χ^2^(1) = 6.46, *p* = .011), matching the nonRT trends for these categories. The only other trend found was an upward trend in links to government or public health websites (χ^2^(1) = 11.77, *p* = .001). This trend was unique to RTs. Comparisons between longitudinal graphs of RT and nonRT results for automated queries found only minor tweet volume changes in a few concepts. A small number of tweet activity spikes in personal opinions/interest, downplayed risk, and misinformation increased in volume when RTs were included.

## Discussion

### Knowledge Translation

The proportion of tweets using the term “H1N1” increased compared to the relative usage of “swine flu”, demonstrating gradual adoption of WHO-recommended terminology by the public and media on Twitter. With some exceptions (#oink campaign, see above) it is likely that the media's and not the public's adoption of “H1N1” was the primary reason for this trend. However, the importance of the media's terminology choice cannot be underestimated as they hold much influence as major information transmitters and word choice is critical in encouraging or discouraging certain risk behaviours [21].

### Tweet Content & Public Attention

In our manual coding we found that news and information were the most commonly tweeted H1N1-related material (52.6%). Our results correspond to a study of Twitter use during Hurricane Gustav and Ike, where roughly half of all hurricane-related tweets contained URLs (web-resources) [22]. Collectively, our findings highlight the role of social networking tools in rapid, widespread communication in emergencies.

The change of tweet content over our timeframe is not unexpected. H1N1 surveys reporting longitudinal results using traditional methods also found that public behaviour and attitudes varied over the course of the epidemic. In these studies, public concern and engagement in protective behaviours increased when the threat of the outbreak increased and decreasing when the perceived risk declined [23], [24], [25]. Similarly, we found that personal accounts of H1N1 increased over time, while the amount of humorous comments decreased, possibly due to the increasing perceived seriousness of the situation and/or the declining popularity of the subject.

More minute changes were also observed and were found to be highly influenced by the media and external events. Examples of this included the large spike in tweets that resulted from the WHO pandemic level 6 announcement and the two peaks in personal experiences that coincided with the first and second wave of H1N1 in North America. Similarly, a study on tweets circulated during the 2009 Red River flooding in North America also found that tweet volume related to the emergency increased when the threat was largest [26], indicating that perceived severity and intense news coverage are likely factors that dictate tweet posting activity. It is possible to qualitatively examine tweet content and see what story has captured the online public's attention and what sentiments those stories evoke. Similar to media stories, both viral dissemination of information and Twitter campaigns had a considerable effect on tweet volume and posting behaviour. The use of these techniques and methods may have potential usefulness in public health and should be studied further.

Our retweet analysis found that the only significant difference was that original tweets contained significantly more tweets with personal experiences compared to retweets. This finding indicates that users are not likely to repost another user's status update en mass and there is potentially little interest or perceived benefit in reposting second-hand personal information. Similarly, other studies have shown that retweets must have either broad appeal or provide specific details of local utility to be widely propagated [27]. Consequently, the tweet spikes that decreased when retweets were removed from the automated data provide a likely indicator of stories that had these qualities.

### Information Demands and Sources

During the outbreak, a variety of traditional media sources speculated that misinformation was rampant in social media [28]. However, we classified only 4.5% of manually coded tweets as possible misinformation or speculation. Although this amount ranged from 2.2 to 9.2% across our 9 time points, increasing amounts of misinformation did not occur until August, months after initial media reports. Tracking tweeted misinformation and questions is potentially useful for public health agencies to address information needs of the public and direct online and offline health education initiatives and campaigns. Media monitoring has been used by the CDC to inform risk communication strategies in previous emergencies [29].

It is noteworthy that 90.2% of tweets provided references to information they were providing, allowing others to confirm the trustworthiness of the material. While the majority of these tweets linked to mainstream or local news websites, the proportion of links to secondary news sites (news blogs/feeds/niches, social networks, and other web pages) increased over time, likely due to information supply and demand (more information from major news providers when the topic is popular and vice versa). The lack of critical assessment and evaluation of online health information by consumers is a well-documented problem [30]. Public health and government authorities such as the CDC and WHO were rarely referenced directly by users (1.5% of links). While mentionings of governing bodies were higher due to the proliferation of news headlines quoting or referring to them, direct linking to the authority and its resources was infrequent. An analysis of retweets also found this was the case, although there was a significant upward trend in linking to authorities over time. This unique trend may indicate that users began to recognize the utility of official resources over time.

### Automated Analysis

The majority of our automated queries correlated with the results of our manual coding, suggesting the feasibility for monitoring large amounts of textual data over time. Our automated queries for concern and personal experiences were also positively related to H1N1 incidence rates, as expected, indicating that our findings have external validity. Queries that did not perform well had less defined vocabularies than others and were more difficult to associate with particular expressions. A caveat we identified is that spam and popular news articles that contained key phrases can influence search results and create peaks in activity that may not be reflective of the concept. Our queries were limited to keywords found in the manual coding and variants that the authors could anticipate. These issues emphasize the importance of analysing the overall content of the tweet and the intricacies of building a substantial search vocabulary and to employ more advanced natural language processing methods.

### Methodological Limitations and Advantages

Public attitudes, perceptions, and behaviours during the pandemic have been reported by other studies using traditional survey methods (e.g., [3], [4], [5]). However, there may be practical limitations to directly comparing our results to these accounts. The largest limitation to our approach in this respect is the lack of a well-defined study population. While our database allows us to link a user with any given tweet, it was beyond the scope of this study to retrieve every user profile in order to determine the demographics of our sample. But, the service is predominantly used by Americans, accounting for 50.8% of all users [31]. Approximately 19% of all online American adults use Twitter or a similar application [32]. It is estimated that in the United States, 55% of Twitter users are female, 45% are aged 18–34, 69% are Caucasian, 49% have less than a college degree, and 58% make over $60K a year [33]. These numbers may give us a sense of population demographics; however, those who tweet about H1N1 may not necessarily be representative of the Twitter population, and the Twitter population is not representative of the general population. In addition, because we are potentially sampling across the globe, it is difficult to narrow the study context and compare results with H1N1 studies that report on a certain geographic region (e.g., [34], [35]). This methodological issue is present also in traditional studies that attempt to corroborate their results with papers from different cities or countries [36]. In the future it may be possible to take advantage of geocoding to address this problem and sort tweets based on location. Secondly, certain questions posed to survey respondents may not be completely translatable to a query concept or category, even if numerous search patterns are used. In regards to our sampling, no existing validated sampling method for Twitter has been documented in the literature and the decisions made in our study may not be optimal in all cases. We recognize that it is likely that not all relevant tweets were represented in our tweet database as some tweets may not have included our keywords and used their own terminology to refer to H1N1.

We did not observe large amounts of misinformation in our data, but this may be a conservative estimate as we did not code humourous or confusing posts as misinformation, nor did we take into account the influence of a tweet based on the number of followers a user had. While our estimates were low, we do not know the effect of any amount of misinformation that exists on the internet, particularly when internet sources are archived and indexed in search engines.

Despite these limitations, there are advantages to using infoveillance. Because our method of data collection is continuous and ongoing, the length of our study time frame likely has no survey-based equivalent. Thus far, the existing H1N1 pandemic studies have collected data anywhere from a span of one day [37] to four months [25]. Those with shorter time frames have reported their results in aggregate, and only a handful has presented longitudinal results of selected questions [3], [23], [24], [25]. Although our manual coding was limited to 9 time points of analysis, Infovigil is continuously collecting and analysing data, creating a significant database that captures both large and small shifts in user posting and puts them into perspective within the overall pandemic picture. This methodology may offer complementary insight to traditional survey methods at a more rapid and less costly rate.

### Conclusion

This study illustrates the potential and feasibility of using social media to conduct “infodemiology” studies for public health. H1N1 pandemic-related tweets on Twitter were primarily used to disseminate information from credible sources to the public, but were also a rich source of opinions and experiences. These tweets can be used for near real-time content and sentiment analysis and knowledge translation research, allowing health authorities to become aware of and respond to real or perceived concerns raised by the public. This study included manual classifications and preliminary automated analyses. More advanced semantic processing tools may be used in the future to classify tweets with more precision and accuracy.

## Supporting Information

Table S1 SQL Queries for Automated Tweet Coding & Analysis. SQL syntax for search patterns and keywords used by Infovigil for automated tweet coding and analysis.
(0.14 MB PDF)
