# Supplementary material for: Pandemics in the Age of Twitter: Content Analysis of Tweets during the 2009 H1N1 Outbreak
Source: PLoS One. 2010 Nov 29;5(11):e14118. doi: 10.1371/journal.pone.0014118 (PMC2993925; doi:10.1371/journal.pone.0014118)
Supplement: Table S1 — SQL Queries for Automated Tweet Coding & Analysis. SQL syntax for search patterns and keywords used by Infovigil for automated tweet coding and analysis. (0.14 MB PDF) [file pone.0014118.s001.pdf]

Table S1: SQL Queries for Automated Tweet Coding & Analysis

Content Concepts

**Resources**	SELECT DISTINCT T.title, T.tweetid, T.id, T.publishdate, T.link, T.content, T.updatedate, T.authorname, T.authoruri, W.webcite_id, W.long_url,COUNT(T.title) AS number_hits FROM tweets T LEFT JOIN webcite W ON T.tweetid = W.tweetid WHERE publishdate BETWEEN "2009-05-01 00:00:00" AND "2009-12-31 23:59:59" AND ((LOWER(`title`) LIKE "%swine flu%" OR LOWER(`title`) LIKE "%swineflu%" OR LOWER(`title`) LIKE "%h1n1%")) AND ((LOWER(`title`) NOT LIKE "%rt @%" AND LOWER(`title`) NOT LIKE "%rt@%")) AND ((LOWER(title) LIKE "%http://%") OR (LOWER(title) LIKE "%https://%")) GROUP by title ORDER BY publishdate ASC
**Personal Experiences**	<u>Subconcept: “Personal/direct H1N1 experience”</u><br>SELECT DISTINCT T.title, T.tweetid, T.id, T.publishdate, T.link, T.content, T.updatedate, T.authorname, T.authoruri, W.webcite_id, W.long_url,COUNT(T.title) AS number_hits FROM tweets T LEFT JOIN webcite W ON T.tweetid = W.tweetid WHERE publishdate BETWEEN "2009-05-01 00:00:00" AND "2009-12-31 23:59:59" AND ((LOWER(`title`) LIKE "%swine flu%" OR LOWER(`title`) LIKE "%swineflu%" OR LOWER(`title`) LIKE "%h1n1%")) AND ((LOWER(`title`) NOT LIKE "%rt @%" AND LOWER(`title`) NOT LIKE "%rt@%")) AND ((LOWER(title) LIKE "%I have swine flu%") OR (LOWER(title) LIKE "%I have h1n1%") OR (LOWER(title) LIKE "%I might have swine flu%") OR (LOWER(title) LIKE "%I might have h1n1%") OR (LOWER(title) LIKE "%I might have the swine flu%") OR (LOWER(title) LIKE "%I think i have h1n1%") OR (LOWER(title) LIKE "%i think i have swine flu%") OR (LOWER(title) LIKE "%i think i have the swine flu%") OR (LOWER(title) LIKE "%i think i might have%") OR (LOWER(title) LIKE "%i feel sick%") OR (LOWER(title) LIKE "%i\\\'m coming down with %") OR (LOWER(title) LIKE "%im coming down with%") OR (LOWER(title) LIKE "%i\'m getting sick%") OR (LOWER(title) LIKE "%im getting sick%") OR (LOWER(title) LIKE "%getting tested for swine flu%") OR (LOWER(title) LIKE "%getting tested for h1n1%") OR (LOWER(title) LIKE "%getting checked out for swine flu%") OR (LOWER(title) LIKE "%getting checked out for h1n1%") OR (LOWER(title) LIKE "%going to the doctor%") OR (LOWER(title) LIKE "%went to the doctor%") OR (LOWER(title) LIKE "%going to see the doctor%") OR (LOWER(title) LIKE "%going to a clinic%") OR (LOWER(title) LIKE "%going to the clinic%") OR (LOWER(title) LIKE "%went to the clinic%") OR (LOWER(title) LIKE "%went to a clinic%") OR (LOWER(title) LIKE "%i have symptoms%") OR (LOWER(title) LIKE "%i have a fever%") OR (LOWER(title) LIKE "%i\'m feeling sick%")) GROUP by title ORDER BY publishdate ASC<br><br>-OR-<br><br><u>Subconcept: “Indirect Experience”</u><br>SELECT DISTINCT T.title, T.tweetid, T.id, T.publishdate, T.link, T.content, T.updatedate, T.authorname, T.authoruri, W.webcite_id, W.long_url,COUNT(T.title) AS number_hits FROM tweets T LEFT JOIN webcite W ON T.tweetid = W.tweetid WHERE publishdate BETWEEN "2009-05-01 00:00:00" AND "2009-12-31 23:59:59" AND ((LOWER(`title`) LIKE "%swine flu%" OR LOWER(`title`) LIKE "%swineflu%" OR LOWER(`title`) LIKE "%h1n1%"))

	AND ((LOWER(`title`) NOT LIKE "%rt @%" AND LOWER(`title`) NOT LIKE "%rt@%")) AND ((LOWER(title) LIKE "%my mom%") OR (LOWER(title) LIKE "%my mum%") OR (LOWER(title) LIKE "%my mother%") OR (LOWER(title) LIKE "%my dad%") OR (LOWER(title) LIKE "%my father%") OR (LOWER(title) LIKE "%my bro%") OR (LOWER(title) LIKE "%my sis%") OR (LOWER(title) LIKE "%my uncle%") OR (LOWER(title) LIKE "%my aunt%") OR (LOWER(title) LIKE "%my grandm%") OR (LOWER(title) LIKE "%my grandpa%") OR (LOWER(title) LIKE "%my grandfather%") OR (LOWER(title) LIKE "%my cousin%") OR (LOWER(title) LIKE "%my niece%") OR (LOWER(title) LIKE "%my nephew%") OR (LOWER(title) LIKE "%my friend%") OR (LOWER(title) LIKE "%my classmate%") OR (LOWER(title) LIKE "%my neighbour%") OR (LOWER(title) LIKE "%my roommate%") OR (LOWER(title) LIKE "%my boyfriend%") OR (LOWER(title) LIKE "%my girlfriend%") OR (LOWER(title) LIKE "%my bf%") OR (LOWER(title) LIKE "%my gf%") OR (LOWER(title) LIKE "%my wife%") OR (LOWER(title) LIKE "%my husband%") OR (LOWER(title) LIKE "%my kid%") OR (LOWER(title) LIKE "%my son%") OR (LOWER(title) LIKE "%my daughter%") OR (LOWER(title) LIKE "%my baby%") OR (LOWER(title) LIKE "%my doctor%") OR (LOWER(title) LIKE "%my co-worker%") OR (LOWER(title) LIKE "%my coworker%") OR (LOWER(title) LIKE "%my co worker%") OR (LOWER(title) LIKE "%my class%") OR (LOWER(title) LIKE "%my school%") OR (LOWER(title) LIKE "%my university%") OR (LOWER(title) LIKE "%my church%") OR (LOWER(title) LIKE "%my city%") OR (LOWER(title) LIKE "%my town%") OR (LOWER(title) LIKE "%my dorm%") OR (LOWER(title) LIKE "%my rez%") OR (LOWER(title) LIKE "%my campus%") OR (LOWER(title) LIKE "%my home%") OR (LOWER(title) LIKE "%my house%") OR (LOWER(title) LIKE "%my office%") OR (LOWER(title) LIKE "%my work%") OR (LOWER(title) LIKE "%my country%")) GROUP by title ORDER BY publishdate ASC<br><br>-OR-<br><br><u>Subconcept: “Vaccination Experience”</u><br>SELECT DISTINCT T.title, T.tweetid, T.id, T.publishdate, T.link, T.content, T.updatedate, T.authorname, T.authoruri, W.webcite_id, W.long_url,COUNT(T.title) AS number_hits FROM tweets T LEFT JOIN webcite W ON T.tweetid = W.tweetid WHERE publishdate BETWEEN "2009-05-01 00:00:00" AND "2009-12-31 23:59:59" AND ((LOWER(`title`) LIKE "%swine flu%" OR LOWER(`title`) LIKE "%swineflu%" OR LOWER(`title`) LIKE "%h1n1%")) AND ((LOWER(`title`) NOT LIKE "%rt @%" AND LOWER(`title`) NOT LIKE "%rt@%")) AND ((LOWER(title) LIKE "%got my shot%") OR (LOWER(title) LIKE "%got my h1n1 shot%") OR (LOWER(title) LIKE "%got my swine flu shot%") OR (LOWER(title) LIKE "%got vaccinated %") OR (LOWER(title) LIKE "%i\'m vaccinated%") OR (LOWER(title) LIKE "%got the swine flu shot%") OR (LOWER(title) LIKE "%got the h1n1 shot%") OR (LOWER(title) LIKE "%got the h1n1 jab%") OR (LOWER(title) LIKE "%got the swine flu jab%") OR (LOWER(title) LIKE "%got my swine flu jab%") OR (LOWER(title) LIKE "%got my h1n1 jab%") OR (LOWER(title) LIKE "%got the h1n1 vaccine%") OR (LOWER(title) LIKE "%got the swine flu vaccine%") OR (LOWER(title) LIKE "%got my swine flu vaccine%") OR (LOWER(title) LIKE "%got my h1n1 vaccine%") OR (LOWER(title) LIKE "%got my h1n1

	immuni%") OR (LOWER(title) LIKE "%got my swine flu immuni%") OR (LOWER(title) LIKE "%went to get the vaccine%") OR (LOWER(title) LIKE "%went to get vaccinated%") OR (LOWER(title) LIKE "%went to get the swine flu shot%") OR (LOWER(title) LIKE "%went to get the swine flu vaccine%") OR (LOWER(title) LIKE "%went to get the swine flu jab%") OR (LOWER(title) LIKE "%went to get my swine flu jab%") OR (LOWER(title) LIKE "%went to get my swine flu shot%") OR (LOWER(title) LIKE "%went to get my swine flu vaccine%") OR (LOWER(title) LIKE "%went to get the h1n1 shot%") OR (LOWER(title) LIKE "%went to get the h1n1 vaccine%") OR (LOWER(title) LIKE "%went to get the h1n1 jab%") OR (LOWER(title) LIKE "%is immuni%") OR (LOWER(title) LIKE "%went to get my h1n1 shot%") OR (LOWER(title) LIKE "%went to get my h1n1 vaccin%") OR (LOWER(title) LIKE "%went to get my h1n1 jab%")) GROUP by title ORDER BY publishdate ASC
**Personal Opinion/Interest**	SELECT DISTINCT T.title, T.tweetid, T.id, T.publishdate, T.link, T.content, T.updatedate, T.authorname, T.authoruri, W.webcite_id, W.long_url,COUNT(T.title) AS number_hits FROM tweets T LEFT JOIN webcite W ON T.tweetid = W.tweetid WHERE publishdate BETWEEN "2009-05-01 00:00:00" AND "2009-12-31 23:59:59" AND ((LOWER(`title`) LIKE "%swine flu%" OR LOWER(`title`) LIKE "%swineflu%" OR LOWER(`title`) LIKE "%h1n1%")) AND ((LOWER(`title`) NOT LIKE "%rt @%" AND LOWER(`title`) NOT LIKE "%rt@%")) AND ((LOWER(title) LIKE "%in my opinion%") OR (LOWER(title) LIKE "%i think%") OR (LOWER(title) LIKE "%imho%") OR (LOWER(title) LIKE "%government should%") OR (LOWER(title) LIKE "%gov\'t should%") OR (LOWER(title) LIKE "%doctors should%") OR (LOWER(title) LIKE "%Obama should%") OR (LOWER(title) LIKE "%media should%") OR (LOWER(title) LIKE "%cdc should%") OR (LOWER(title) LIKE "%i believe%") OR (LOWER(title) LIKE "%schools should%") OR (LOWER(title) LIKE "%teachers should%") OR (LOWER(title) LIKE "%hospitals should%") OR (LOWER(title) LIKE "%people should%") OR (LOWER(title) LIKE "%my stance%") OR (LOWER(title) LIKE "%my take%") OR (LOWER(title) LIKE "%my view%") OR (LOWER(title) LIKE "%my feeling%") OR (LOWER(title) LIKE "%my impression%") OR (LOWER(title) LIKE "%my theory%") OR (LOWER(title) LIKE "%my thought%") OR (LOWER(title) LIKE "% pov %") OR (LOWER(title) LIKE "%my opinion%") OR (LOWER(title) LIKE "%i recommend%") OR (LOWER(title) LIKE "%i suggest%") OR (LOWER(title) LIKE "%my suggestion%") OR (LOWER(title) LIKE "%did you hear%") OR (LOWER(title) LIKE "%reading%" AND NOT LOWER(title) LIKE "%spreading%") OR (LOWER(title) LIKE "%interest%") OR (LOWER(title) LIKE "%looking up%" AND NOT LOWER(title) LIKE "%things are looking up%" AND NOT LOWER(title) LIKE "%it\'slooking up%") OR (LOWER(title) LIKE "%researching%") OR (LOWER(title) LIKE "%heard %") OR (LOWER(title) LIKE "%read %" AND NOT LOWER(title) LIKE "%spread %" OR LOWER(title) LIKE "%I read %") OR (LOWER(title) LIKE "%i hear %") OR (LOWER(title) LIKE "%i feel that%")) GROUP by title ORDER BY publishdate ASC

Qualifiers Concepts

**Humour/Sarcasm**	SELECT DISTINCT T.title, T.tweetid, T.id, T.publishdate, T.link, T.content, T.updatedate, T.authorname, T.authoruri, W.webcite_id, W.long_url,COUNT(T.title) AS number_hits FROM tweets T LEFT JOIN webcite W ON T.tweetid = W.tweetid WHERE publishdate BETWEEN "2009-05-01 00:00:00" AND "2009-12-31 23:59:59" AND ((LOWER(\`title\`) LIKE "%swine flu%" OR LOWER(\`title\`) LIKE "%swineflu%" OR LOWER(\`title\`) LIKE "%h1n1%")) AND ((LOWER(\`title\`) NOT LIKE "%rt @%" AND LOWER(\`title\`) NOT LIKE "%rt@%")) AND ((LOWER(title) LIKE "%lol%") OR (LOWER(title) LIKE "%LMAO%") OR (LOWER(title) LIKE "%haha%") OR (LOWER(title) LIKE "%hehe%") OR (LOWER(title) LIKE "%hilarious%") OR (LOWER(title) LIKE "%funny%") OR (LOWER(title) LIKE "%ode to tamiflu%") OR (LOWER(title) LIKE "%j/k%") OR (LOWER(title) LIKE "% XD %") OR (LOWER(title) LIKE "%:P%") OR (LOWER(title) LIKE "%=P%") OR (LOWER(title) LIKE "%ROFL%") OR (LOWER(title) LIKE "%:)%") OR (LOWER(title) LIKE "%=)%") OR (LOWER(title) LIKE "% jk %") OR (LOWER(title) LIKE "%xP %") OR (LOWER(title) LIKE "%;)%") OR (LOWER(title) LIKE "%=D%") OR (LOWER(title) LIKE "%:D%") OR (LOWER(title) LIKE "%ha ha%") OR (LOWER(title) LIKE "%joking%") OR (LOWER(title) LIKE "%just kiddin%") OR (LOWER(title) LIKE "%piggy%") OR (LOWER(title) LIKE "%oink%" AND NOT LOWER(title) LIKE "%#oink%") OR (LOWER(title) LIKE "%bacon flu%") OR (LOWER(title) LIKE "%when pigs fly%") OR (LOWER(title) LIKE "%he he %") OR (LOWER(title) LIKE "%heh%") OR (LOWER(title) LIKE "%unlikelysequels%") OR (LOWER(title) LIKE "%bacon fever%") OR (LOWER(title) LIKE "%joke%" AND NOT LOWER(title) LIKE "%what a joke%") OR (LOWER(title) LIKE "%hiney%") OR (LOWER(title) LIKE "%heinie%")) GROUP by title ORDER BY publishdate ASC
**Relief**	SELECT DISTINCT T.title, T.tweetid, T.id, T.publishdate, T.link, T.content, T.updatedate, T.authorname, T.authoruri, W.webcite_id, W.long_url,COUNT(T.title) AS number_hits FROM tweets T LEFT JOIN webcite W ON T.tweetid = W.tweetid WHERE publishdate BETWEEN "2009-05-01 00:00:00" AND "2009-12-31 23:59:59" AND ((LOWER(\`title\`) LIKE "%swine flu%" OR LOWER(\`title\`) LIKE "%swineflu%" OR LOWER(\`title\`) LIKE "%h1n1%")) AND ((LOWER(title) NOT LIKE "%RT@%" AND "%RT @%")) AND ((LOWER(title) LIKE "%relieved%") OR (LOWER(title) LIKE "%thank God%") OR (LOWER(title) LIKE "%thankful%") OR (LOWER(title) LIKE "%whew%") OR (LOWER(title) LIKE "%i am ok%") OR (LOWER(title) LIKE "%im ok%") OR (LOWER(title) LIKE "%all better%") OR (LOWER(title) LIKE "%feeling % better%" AND NOT LOWER(title) LIKE "%not feeling % better%") OR (LOWER(title) LIKE "%relief%") OR (LOWER(title) LIKE "%i\\'m recovering%") OR (LOWER(title) LIKE "%i just recovered%") OR (LOWER(title) LIKE "%i\\'ve recovered%") OR (LOWER(title) LIKE "%back at school%") OR (LOWER(title) LIKE "%back at work%") OR (LOWER(title) LIKE "%glad that %") OR (LOWER(title) LIKE "%happy to hear%") OR (LOWER(title) LIKE "%glad to hear%") OR (LOWER(title) LIKE "%i\\'m safe%") OR (LOWER(title) LIKE "%grateful%" AND NOT LOWER(title) LIKE "%ungrateful%") OR (LOWER(title) LIKE "%good to hear%") OR (LOWER(title) LIKE "%happy that%") OR (LOWER(title) LIKE "%good that%") OR (LOWER(title) LIKE "%survived%") OR (LOWER(title) LIKE "%bounced back%") OR (LOWER(title) LIKE "%recuperate%") OR

	(LOWER(title) LIKE "%escape%")) GROUP by title ORDER BY publishdate ASC
**Concern**	Subconcept: “Concern for Others”<br>SELECT DISTINCT T.title, T.tweetid, T.id, T.publishdate, T.link, T.content, T.updatedate, T.authorname, T.authoruri, W.webcite_id, W.long_url,COUNT(T.title) AS number_hits FROM tweets T LEFT JOIN webcite W ON T.tweetid = W.tweetid WHERE publishdate BETWEEN "2009-05-01 00:00:00" AND "2009-12-31 23:59:59" AND ((LOWER(\`title\`) LIKE "%swine flu%" OR LOWER(\`title\`) LIKE "%swineflu%" OR LOWER(\`title\`) LIKE "%h1n1%")) AND ((LOWER(title) NOT LIKE “%RT@%” AND “%RT @%”)) AND ((LOWER(title) LIKE "%get better%") OR (LOWER(title) LIKE "%get well%") OR (LOWER(title) LIKE "%take care%") OR (LOWER(title) LIKE "%are you ok%") OR (LOWER(title) LIKE "%is it swine flu%") OR (LOWER(title) LIKE "%is it h1n1%") OR (LOWER(title) LIKE "%is it the swine flu%") OR (LOWER(title) LIKE "%r u ok%") OR (LOWER(title) LIKE "%hope % is ok%") OR (LOWER(title) LIKE "%hope you don\'t get %") OR (LOWER(title) LIKE "%hope you\'re not sick%") OR (LOWER(title) LIKE "%hope you aren\'t sick%") OR (LOWER(title) LIKE "%hope u r ok%") OR (LOWER(title) LIKE "%hope u don\'t get %") OR (LOWER(title) LIKE "%hope u aren\'t infected%") OR (LOWER(title) LIKE "%hope you aren\'t infected%") OR (LOWER(title) LIKE "%go to the doctor%") OR (LOWER(title) LIKE "%go to the clinic%") OR (LOWER(title) LIKE "%get checked out%") OR (LOWER(title) LIKE "%get tested%") OR (LOWER(title) LIKE "%quarantine yourself%") OR (LOWER(title) LIKE "%stay home%") OR (LOWER(title) LIKE "%has swine flu%") OR (LOWER(title) LIKE "%has h1n1%") OR (LOWER(title) LIKE "%has the swine flu%") OR (LOWER(title) LIKE "%poor %")) GROUP by title ORDER BY publishdate ASC<br><br>-OR-<br><br>Subconcept: “Concerned Emoticons”<br>SELECT DISTINCT T.title, T.tweetid, T.id, T.publishdate, T.link, T.content, T.updatedate, T.authorname, T.authoruri, W.webcite_id, W.long_url,COUNT(T.title) AS number_hits FROM tweets T LEFT JOIN webcite W ON T.tweetid = W.tweetid WHERE publishdate BETWEEN "2009-05-01 00:00:00" AND "2009-12-31 23:59:59" AND ((LOWER(\`title\`) LIKE "%swine flu%" OR LOWER(\`title\`) LIKE "%swineflu%" OR LOWER(\`title\`) LIKE "%h1n1%")) AND ((LOWER(title) NOT LIKE “%RT@%” AND “%RT @%”)) AND ((LOWER(title) LIKE "%:(%") OR (LOWER(title) LIKE "%=(%") OR (LOWER(title) LIKE "%:-(%") OR (LOWER(title) LIKE "%:\'(%") OR (LOWER(title) LIKE "%=\'(%") OR (LOWER(title) LIKE "%:\\%") OR (LOWER(title) LIKE "%=|%") OR (LOWER(title) LIKE "%:|%") OR (LOWER(title) LIKE "%:o%") OR (LOWER(title) LIKE "%o.o%") OR (LOWER(title) LIKE "% :S %")) GROUP by title ORDER BY publishdate ASC<br><br>-OR-<br><br>Subconcept: “General Concern”<br>SELECT DISTINCT T.title, T.tweetid, T.id, T.publishdate, T.link, T.content, T.updatedate, T.authorname, T.authoruri, W.webcite_id, W.long_url,COUNT(T.title) AS number_hits FROM tweets T LEFT JOIN webcite W ON T.tweetid =

	W.tweetid WHERE publishdate BETWEEN "2009-05-01 00:00:00" AND "2009-12-31 23:59:59" AND ((LOWER(`title`) LIKE "%swine flu%" OR LOWER(`title`) LIKE "%swineflu%" OR LOWER(`title`) LIKE "%h1n1%")) AND ((LOWER(title) NOT LIKE “%RT@%” AND “%RT @%”)) AND ((LOWER(title) LIKE "%omg%") OR (LOWER(title) LIKE "%oh my god%") OR (LOWER(title) LIKE "%uh oh%") OR (LOWER(title) LIKE "%oh no%") OR (LOWER(title) LIKE "%worried%" AND NOT LOWER(title) LIKE "%not worried%" AND NOT LOWER(title) LIKE "%unworried%") OR (LOWER(title) LIKE "%scared%" AND NOT LOWER(title) LIKE "%not scared%" AND NOT LOWER(title) LIKE "%don\'t bescared%" AND NOT LOWER(title) LIKE "%dont be scared%") OR (LOWER(title) LIKE "%stay away%") OR (LOWER(title) LIKE "%dangerous%" AND NOT LOWER(title) LIKE "%not dangerous%" AND NOT LOWER(title) LIKE "%no more dangerous%" AND NOT LOWER(title) LIKE "%less dangerous%") OR (LOWER(title) LIKE "%terrified%") OR (LOWER(title) LIKE "%afraid%" AND NOT LOWER(title) LIKE "%not afraid%" AND NOT LOWER(title) LIKE "%don\'t be afraid%" AND NOT LOWER(title) LIKE "%stop being afraid%" AND NOT LOWER(title) LIKE "%unafraid%" AND NOT LOWER(title) LIKE "%dont be afraid%") OR (LOWER(title) LIKE "%frightened%" AND NOT LOWER(title) LIKE "%not frightened%") OR (LOWER(title) LIKE "%freaking out%" AND NOT LOWER(title) LIKE "%not freaking out%" AND NOT LOWER(title) LIKE "%stop freaking out%") OR (LOWER(title) LIKE "%freakin out%" AND NOT LOWER(title) LIKE "%not freakin out%" AND NOT LOWER(title) LIKE "%stop freakin out%") OR (LOWER(title) LIKE "%nervous%") OR (LOWER(title) LIKE "%sad%" AND NOT LOWER(title) LIKE "%palisad%" AND NOT LOWER(title) LIKE "%passad%" AND NOT LOWER(title) LIKE "%pasad%") OR (LOWER(title) LIKE "%grief%") OR (LOWER(title) LIKE "%stress%" AND NOT LOWER(title) LIKE "%mistress%" AND NOT LOWER(title) LIKE "%officials stress%") OR (LOWER(title) LIKE "%scary%" AND NOT LOWER(title) LIKE "%not scary%") OR (LOWER(title) LIKE "%confused%") OR (LOWER(title) LIKE "%confusing%") OR (LOWER(title) LIKE "%yikes%") OR (LOWER(title) LIKE "%uneasy%") OR (LOWER(title) LIKE "%I worry%") OR (LOWER(title) LIKE "%worrisome%" AND NOT LOWER(title) LIKE "%not worrisome%") OR (LOWER(title) LIKE "%dread%") OR (LOWER(title) LIKE "%concern%" AND NOT LOWER(title) LIKE "%unconcern%" AND NOT LOWER(title) LIKE "%not concern%" AND NOT LOWER(title) LIKE "%don\'t be concern%" AND NOT LOWER(title) LIKE "%dont be concern%" AND NOT LOWER(title) LIKE "%don\'t concern%" AND NOT LOWER(title) LIKE "%dont concern%" OR LOWER(title) LIKE "%i am concern%" OR LOWER(title) LIKE "%i\\\'m concern%" OR LOWER(title) LIKE "%im concern%" OR LOWER(title) LIKE "%kind of concern%" OR LOWER(title) LIKE "%kinda concern%" OR LOWER(title) LIKE "%sort of concern%" OR LOWER(title) LIKE "%sorta concern%" OR LOWER(title) LIKE "%really concern%")) GROUP by title ORDER BY publishdate ASC<br><br>-OR-<br><br><u>Subconcept: “Concern for Self”</u><br>SELECT DISTINCT T.title, T.tweetid, T.id, T.publishdate, T.link, T.content, T.updatedate, T.authorname, T.authoruri, W.webcite_id, W.long_url,COUNT(T.title) AS number_hits FROM tweets T LEFT JOIN webcite W ON T.tweetid =

	W.tweetid WHERE publishdate BETWEEN "2009-05-01 00:00:00" AND "2009-12-31 23:59:59" AND ((LOWER(`title`) LIKE "%swine flu%" OR LOWER(`title`) LIKE "%swineflu%" OR LOWER(`title`) LIKE "%h1n1%")) AND ((LOWER(title) NOT LIKE "%RT@%" AND "%RT @%")) AND ((LOWER(title) LIKE "%I might have h1n1%") OR (LOWER(title) LIKE "%I might have swine flu%") OR (LOWER(title) LIKE "%I might have the swine flu%") OR (LOWER(title) LIKE "%I think I have the swine flu%") OR (LOWER(title) LIKE "%I think I have swine flu%") OR (LOWER(title) LIKE "%I think I have h1n1%") OR (LOWER(title) LIKE "%I don\'t want to die%") OR (LOWER(title) LIKE "%I have a fever%") OR (LOWER(title) LIKE "%I have symptoms%") OR (LOWER(title) LIKE "%I feel sick%") OR (LOWER(title) LIKE "%not feeling well%") OR (LOWER(title) LIKE "%feeling sick%") OR (LOWER(title) LIKE "%I\'m sick%")) GROUP by title ORDER BY publishdate ASC
**Downplay**	SELECT DISTINCT T.title, T.tweetid, T.id, T.publishdate, T.link, T.content, T.updatedate, T.authorname, T.authoruri, W.webcite_id, W.long_url,COUNT(T.title) AS number_hits FROM tweets T LEFT JOIN webcite W ON T.tweetid = W.tweetid WHERE publishdate BETWEEN "2009-05-01 00:00:00" AND "2009-12-31 23:59:59" AND ((LOWER(`title`) LIKE "%swine flu%" OR LOWER(`title`) LIKE "%swineflu%" OR LOWER(`title`) LIKE "%h1n1%")) AND ((LOWER(`title`) NOT LIKE "%rt @%" AND LOWER(`title`) NOT LIKE "%rt@%")) AND ((LOWER(title) LIKE "%not a big deal%") OR (LOWER(title) LIKE "%hype%") OR (LOWER(title) LIKE "%overblown%") OR (LOWER(title) LIKE "%just swine flu%") OR (LOWER(title) LIKE "%not worried%") OR (LOWER(title) LIKE "%not afraid%") OR (LOWER(title) LIKE "%forget about swine flu%" AND NOT LOWER(title) LIKE "%don\'t forget about swine flu%" AND NOT LOWER(title) LIKE "%dont forget about swine flu%" AND NOT LOWER(title) LIKE "%do not forget about swine flu%") OR (LOWER(title) LIKE "%it\\\'s not that bad%") OR (LOWER(title) LIKE "%i\\\'ve had worse%") OR (LOWER(title) LIKE "%hysteria%") OR (LOWER(title) LIKE "%just relax%") OR (LOWER(title) LIKE "%calm down%") OR (LOWER(title) LIKE "%don\'t panic%") OR (LOWER(title) LIKE "%paranoi%" AND NOT LOWER(title) LIKE "%I am paranoi%" AND NOT LOWER(title) LIKE "%Im paranoi%" AND NOT LOWER(title) LIKE "%I\'m paranoi%" AND NOT LOWER(title) LIKE "%I\'m getting paranoi%" AND NOT LOWER(title) LIKE "%Im getting paranoi%" AND NOT LOWER(title) LIKE "%I am paranoi%" AND NOT LOWER(title) LIKE "%I\'m becoming paranoi%" AND NOT LOWER(title) LIKE "%I am becoming paranoi%" AND NOT LOWER(title) LIKE "%Im becomingparanoi%" AND NOT LOWER(title) LIKE "%I am so paranoi%" AND NOT LOWER(title) LIKE "%I\'m so paranoi%" AND NOT LOWER(title) LIKE "%Im so paranoi%") OR (LOWER(title) LIKE "%not concerned%") OR (LOWER(title) LIKE "%like regular flu%") OR (LOWER(title) LIKE "%what\'s the big deal%") OR (LOWER(title) LIKE "%milder than regular flu%") OR (LOWER(title) LIKE "%not dangerous%") OR (LOWER(title) LIKE "%less dangerous%") OR (LOWER(title) LIKE "%less deadly%") OR (LOWER(title) LIKE "%milder than seasonal%") OR (LOWER(title) LIKE "%more people die from%") OR (LOWER(title) LIKE "%million people have aids%") OR (LOWER(title) LIKE "%forget about h1n1%" AND NOT LOWER(title) LIKE "%don\'t forget about h1n1%" AND NOT LOWER(title) LIKE "%dont forget about h1n1%" AND NOT LOWER(title) LIKE "%do not forget about h1n1%") OR (LOWER(title) LIKE "%don\'t care%")

<table>
<tr><td></td><td>OR (LOWER(title) LIKE "%not worried%") OR (LOWER(title) LIKE "%dont care%") OR (LOWER(title) LIKE "%who cares%") OR (LOWER(title) LIKE "%forget swine flu%" AND NOT LOWER(title) LIKE "%don\'t forget swine flu%" AND NOT LOWER(title) LIKE "%dont forget swine flu%" AND NOT LOWER(title) LIKE "%do not forget swine flu%" AND NOT LOWER(title) LIKE "%won\'t forget swine flu%") OR (LOWER(title) LIKE "%forget h1n1%" AND NOT LOWER(title) LIKE "%don\'t forget h1n1%" AND NOT LOWER(title) LIKE "%dont forget h1n1%" AND NOT LOWER(title) LIKE "%do not forget h1n1%") OR (LOWER(title) LIKE "%swine flu is nothing%") OR (LOWER(title) LIKE "%h1n1 is nothing%") OR (LOWER(title) LIKE "%like normal flu%") OR (LOWER(title) LIKE "%like regular flu%") OR (LOWER(title) LIKE "%like seasonal flu%")) GROUP by title ORDER BY publishdate ASC</td></tr>
<tr><td>Frustration</td><td>SELECT DISTINCT T.title, T.tweetid, T.id, T.publishdate, T.link, T.content, T.updatedate, T.authorname, T.authoruri, W.webcite_id, W.long_url,COUNT(T.title) AS number_hits FROM tweets T LEFT JOIN webcite W ON T.tweetid = W.tweetid WHERE publishdate BETWEEN "2009-05-01 00:00:00" AND "2009-12-31 23:59:59" AND ((LOWER(`title`) LIKE "%swine flu%" OR LOWER(`title`) LIKE "%swineflu%" OR LOWER(`title`) LIKE "%h1n1%")) AND ((LOWER(`title`) NOT LIKE "%rt @%" AND LOWER(`title`) NOT LIKE "%rt@%")) AND ((LOWER(title) LIKE "%swine flu sucks%") OR (LOWER(title) LIKE "%grr%%") OR (LOWER(title) LIKE "%WTF%") OR (LOWER(title) LIKE "%annoy%") OR (LOWER(title) LIKE "%irritat%") OR (LOWER(title) LIKE "%pathetic%") OR (LOWER(title) LIKE "%pissed%") OR (LOWER(title) LIKE "%freakin swine flu%") OR (LOWER(title) LIKE "%FML%") OR (LOWER(title) LIKE "%fuck%") OR (LOWER(title) LIKE "%shit%") OR (LOWER(title) LIKE "%:@%") OR (LOWER(title) LIKE "%>:|%") OR (LOWER(title) LIKE "%>:(%") OR (LOWER(title) LIKE "%shut%up%") OR (LOWER(title) LIKE "%friggin%") OR (LOWER(title) LIKE "%getting out of hand%") OR (LOWER(title) LIKE "%stupid%") OR (LOWER(title) LIKE "%hate swine flu%") OR (LOWER(title) LIKE "%hate h1n1%") OR (LOWER(title) LIKE "%hate being sick%") OR (LOWER(title) LIKE "%hate it%") OR (LOWER(title) LIKE "%h1n1 sucks%") OR (LOWER(title) LIKE "%damn%") OR (LOWER(title) LIKE "%effing%") OR (LOWER(title) LIKE "%freaking swine flu%") OR (LOWER(title) LIKE "%frick%") OR (LOWER(title) LIKE "%freaking h1n1%") OR (LOWER(title) LIKE "%freakin h1n1%") OR (LOWER(title) LIKE "%i can\'t believe%") OR (LOWER(title) LIKE "%they better not%") OR (LOWER(title) LIKE "%I am mad %" OR LOWER(title) LIKE "%I\'m mad %" OR LOWER(title) LIKE "%Im mad %" OR LOWER(title) LIKE "%is mad %" OR LOWER(title) LIKE "%so mad %") OR (LOWER(title) LIKE "%frustrated%") OR (LOWER(title) LIKE "%angry%") OR (LOWER(title) LIKE "%outrage%") OR (LOWER(title) LIKE "%cranky%") OR (LOWER(title) LIKE "%peeved%") OR (LOWER(title) LIKE "%furious%") OR (LOWER(title) LIKE "%bitter%") OR (LOWER(title) LIKE "% irk%") OR (LOWER(title) LIKE "%crushed%") OR (LOWER(title) LIKE "%so sick of%") OR (LOWER(title) LIKE "%if i hear%")) GROUP by title ORDER BY publishdate ASC</td></tr>
<tr><td>Misinformation</td><td>SELECT DISTINCT T.title, T.tweetid, T.id, T.publishdate, T.link, T.content, T.updatedate, T.authorname, T.authoruri, W.webcite_id, W.long_url,COUNT(T.title) AS number_hits FROM tweets T LEFT JOIN webcite W ON T.tweetid = W.tweetid WHERE publishdate BETWEEN "2009-05-01 00:00:00" AND "2009-</td></tr>
</table>

	12-31 23:59:59"AND ((LOWER(`title`) LIKE "%swine flu%" OR LOWER(`title`) LIKE "%swineflu%" OR LOWER(`title`) LIKE "%h1n1%")) AND ((LOWER(`title`) NOT LIKE "%rt @%" AND LOWER(`title`) NOT LIKE "%rt@%")) AND ((LOWER(title) LIKE "%hidden%") OR (LOWER(title) LIKE "%secret %") OR (LOWER(title) LIKE "%conspiracy%" AND NOT LOWER(title) LIKE "%not a conspiracy%") OR (LOWER(title) LIKE "%conspirator%") OR (LOWER(title) LIKE "%autism%") OR (LOWER(title) LIKE "%hoax%") OR (LOWER(title) LIKE "%apocalypse%") OR (LOWER(title) LIKE "%armageddon%") OR (LOWER(title) LIKE "%real story%") OR (LOWER(title) LIKE "%poison%") OR (LOWER(title) LIKE "%guillain-barre%") OR (LOWER(title) LIKE "%eugenics%") OR (LOWER(title) LIKE "%plot%") OR (LOWER(title) LIKE "%mind control%") OR (LOWER(title) LIKE "%weapon%") OR (LOWER(title) LIKE "%bioterrorism%") OR (LOWER(title) LIKE "%chemical warfare%") OR (LOWER(title) LIKE "%toxin%") OR (LOWER(title) LIKE "%toxic%") OR (LOWER(title) LIKE "% lie %") OR (LOWER(title) LIKE "% lies %") OR (LOWER(title) LIKE "%mindcontrol%") OR (LOWER(title) LIKE "%mind-control%")) GROUP by title ORDER BY publishdate ASC
**Question**	SELECT DISTINCT T.title, T.tweetid, T.id, T.publishdate, T.link, T.content, T.updatedate, T.authorname, T.authoruri, W.webcite_id, W.long_url,COUNT(T.title) AS number_hits FROM tweets T LEFT JOIN webcite W ON T.tweetid = W.tweetid WHERE publishdate BETWEEN "2009-05-01 00:00:00" AND "2009-12-31 23:59:59" AND ((LOWER(`title`) LIKE "%swine flu%" OR LOWER(`title`) LIKE "%swineflu%" OR LOWER(`title`) LIKE "%h1n1%")) AND AND ((LOWER(`title`) NOT LIKE "%rt @%" AND LOWER(`title`) NOT LIKE "%rt@%")) ((LOWER(title) LIKE "%?%")) GROUP by title ORDER BY publishdate ASC
